# Selenium–Chondroitin Sulfate Nanoparticles Inhibit Angiogenesis by Regulating the VEGFR2-Mediated PI3K/Akt Pathway

**DOI:** 10.3390/md23010022

**Published:** 2025-01-02

**Authors:** Xia Zheng, Xiaofei Liu, Zhuo Wang, Rui Li, Qiaoli Zhao, Bingbing Song, Kit-Leong Cheong, Jianping Chen, Saiyi Zhong

**Affiliations:** 1 College of Food Science and Technology, Guangdong Ocean University, Zhanjiang 524088, China; zx18723218830@126.com (X.Z.); liuxf169@126.com (X.L.); wangzhuo4132@outlook.com (Z.W.); liruihn@163.com (R.L.); zql2819557995@163.com (Q.Z.); 15891793858@163.com (B.S.); klcheong@stu.edu.cn (K.-L.C.); 2 Collaborative Innovation Center of Seafood Deep Processing, Dalian Polytechnic University, Dalian 116034, China; 3 Guangdong Provincial Key Laboratory of Aquatic Product Processing and Safety, Zhanjiang 524088, China; 4 Guangdong Provincial Engineering Technology Research Center of Seafood, Zhanjiang 524088, China; 5 Guangdong Province Engineering Laboratory for Marine Biological Products, Zhanjiang 524088, China; 6 Guangdong Provincial Modern Agricultural Science and Technology Innovation Center for Subtropical Fruit and Vegetable Processing, Zhanjiang 524088, China; 7 Guangdong Provincial Key Laboratory of Intelligent Food Manufacturing, Foshan University, Foshan 528000, China

**Keywords:** selenium–chondroitin sulfate, human umbilical vein endothelial cells, chick embryo chorionic allantoic assay, angiogenesis, VEGFR2 protein

## Abstract

Chondroitin sulfate (CS), a class of glycosaminoglycans covalently attached to proteins to form proteoglycans, is widely distributed in the extracellular matrix and cell surface of animal tissues. In our previous study, CS was used as a template for the synthesis of seleno-chondroitin sulfate (SeCS) through the redox reaction of ascorbic acid (Vc) and sodium selenite (Na_2_SeO_3_) and we found that SeCS could inhibit tumor cell proliferation and invasion. However, its effect on angiogenesis and its underlying mechanism are unknown. In this study, we analyzed the effect of SeCS on tube formation in vitro, based on the inhibition of tube formation and migration of human umbilical vein endothelial cells (HUVECs), and evaluated the in vivo angiogenic effect of SeCS using the chick embryo chorioallantoic membrane (CAM) assay. The results showed that SeCS significantly inhibited the angiogenesis of chicken embryo urothelium. Further mechanism analysis showed that SeCS had a strong inhibitory effect on VEGFR2 expression and its downstream PI3K/Akt signaling pathway, which contributed to its anti-angiogenic effects. In summary, SeCS showed good anti-angiogenic effects in an HUVEC cell model and a CAM model, suggesting that it may be a potential angiogenesis inhibitor.

## 1. Introduction

Angiogenesis refers to the process in which vascular endothelial cells generate new capillary networks through migration, proliferation, the degeneration of basement membrane, matrix remodeling and lumen formation [1], and the angiogenesis is closely related to tumor growth, recurrence and metastasis, which is mainly due to the proliferation of the vascular network within the tumor to provide nutrients and oxygen for the tumor to ensure tumor growth [2,3]. Moreover, under the action of various growth factors released by the tumor, angiogenesis further promotes tumor proliferation and infiltration [4]. Therefore, anti-angiogenesis has become an important way to cut off the malignant growth of tumors and block tumor invasion and metastasis, which can effectively curb the proliferation of tumors and is an effective strategy for the treatment of tumors [5,6]. However, current tumor angiogenesis inhibitors such as Bevacizumab (Bev) and Resumumab often lead to serious side effects [7]. With prolonged treatment, tumor cells may develop drug resistance, leading to a decrease in drug efficacy [8], resulting in high blood pressure [9], and the limited effectiveness of a single treatment [10]. Thus, the development of new anti-vascular drugs has become a hot research topic in recent years. Kung et al. demonstrated that, in endothelial and hepatocellular carcinoma cells, ginger oleoresinone nanoparticles significantly inhibited the onset of tumor angiogenesis by inhibiting the VEGF-mediated PI3K/Akt/mTOR signaling pathway and Akt/eNOS signaling [11]. Wang et al. demonstrated that ethyl cinnamate could inhibit tumor growth through the VEGFR2 signaling pathway to inhibit angiogenesis [12]. Fu et al. found that apigenin had a strong inhibitory effect on HIF-1 α expression and its downstream VEGF-A/VEGFR2 and PDGF-BB/PDGFβR signaling pathways, which in turn inhibited tumor angiogenesis [7].

**Figure 1:**
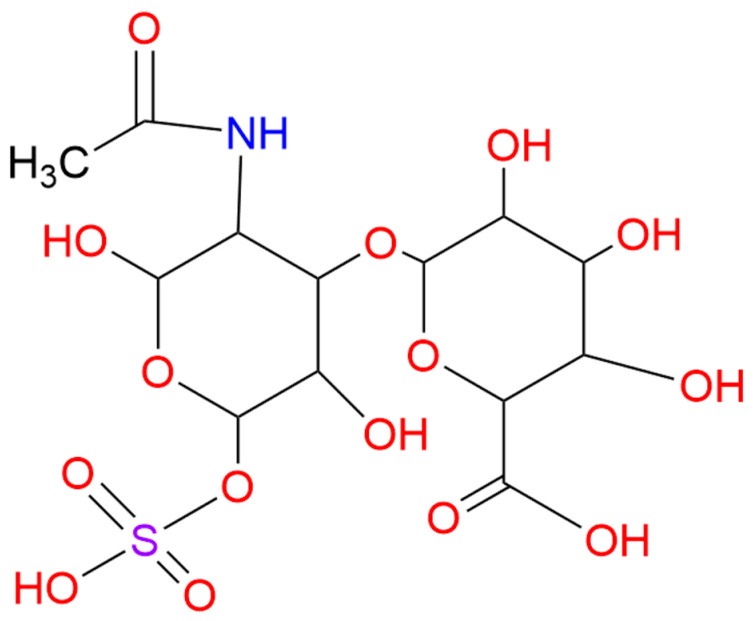
Molecular structure of CS from shark.

Selenium is an imperative trace element for the human body. Selenium nanoparticles (SeNPs) have the advantages of high surface energy, small size and large specific surface area, thus promoting cellular uptake and improving their biological activity [13,14]. However, the easy deactivation of red selenium nanoparticle monomers has limited their utilization in food, drug and nutraceutical research [15]. Therefore, the selection of appropriate stabilizers and dispersants is pivotal. Chondroitin sulfate (CS), a natural polymer of β-glucuronic acid-(1→3)N-acetyl-β-galactosamine [β-GlcA-(1→3)-GalNAc-(1→4)] (Figure 1), is odorless, hygroscopic and viscous when in contact with water and is widely found in animal tissues, cell surfaces and cell matrices and CS is widely used as a dietary protective agent [16,17]. Studies have shown that CS from *Litopenaeus vannamei* shrimp could inhibit melanoma cell migration and endothelial cell tube formation [18]. Moreover, its modifiers also could suppress endothelial cell proliferation and induced cell apoptosis [19]. In our previous study, seleno-chondroitin sulfate (SeCS) was prepared and we found that it possessed anti-tumor effects [20,21]. However, the effect of SeCS on tumor angiogenesis remains unclear.

Human umbilical vein endothelial cells (HUVECs) are derived from the umbilical cord tissue of newborns and have the advantages of being easy to obtain, having no ethical controversies and making it easy to extract cells [22]. Moreover, HUVECs have the ability of cellular tube formation and are the main cell model for studying the function of vascular endothelial cells in vitro. Zhang et al. used the HUVECs model to examine the effect of Jujuboside B on the vasculature of human colon carcinoma HCT-15 [23]. In this study, HUVECs were used to mimic the state of tumor vascular endothelial cells and the effects of SeCS on the proliferation, migration, invasion and lumen formation of HUVECs were evaluated. Meanwhile, the in vivo angiogenic effects of SeCS were investigated using the CAM assay and its underlying molecular mechanism was elucidated using Western blot assays. This study demonstrates that SeCS has the potential to become a new anti-tumor angiogenesis inhibitor in the treatment of cancer.

## 2. Results and Discussion

### 2.1. Role of SeCS in the Tube Formation of HUVECs

**Figure 2:**
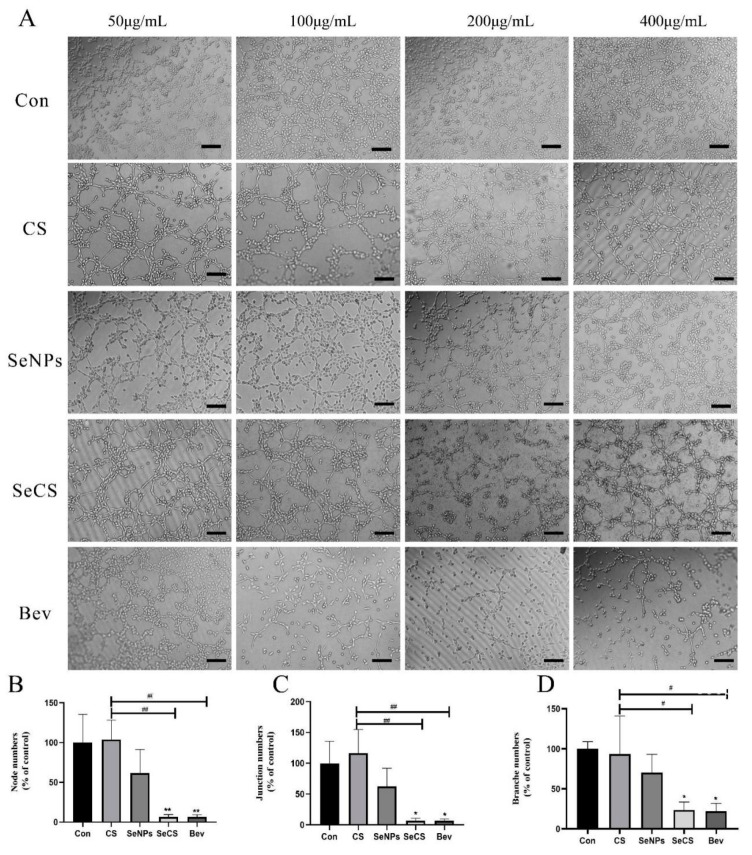
Effects of different treatments on tube formation. (**A**) Concentration-dependent vascular disruption process of CS, SeNPs, SeCS and Bev incubated with blood vessels for a 6 h period at 37 °C. (**B**) The generation rate for the number of primary nodes produced by different groups at a concentration of 200 µg/mL. (**C**) The number of junctions and the generation rate for each group at 200 µg/mL. (**D**) The total lumen branch length and the generation rate in diverse groups at 200 µg/mL. * *p* < 0.05 and ** *p* < 0.01 compared to the control group, respectively. ^#^ *p* < 0.05 and ^##^ *p* < 0.01 compared with each other of sample groups. Scale bars = 100 μm.

Angiogenesis can be divided into the following four stages: first, the production of protease degrades the basement membrane; then, endothelial cell activation and migration occur; subsequently, proliferation occurs; finally, tubular structures and capillaries are formed [24]. HUVECs are a single layer of cells lining blood vessels and play an important role in maintaining the structure and function of blood vessels [25]. Therefore, detecting the number of HUVEC tubes formed can reflect the effect of SeCS on early angiogenesis in vitro. The results are shown in Figure 2A. Notable suppression of tube formation was observed with both Bev and SeCS across all dosage groups, with the extent of inhibition varying with changes in concentration. The anti-angiogenic effect of SeCS at 200 µg/mL was greater than that of CS and SeNPs; in addition, HUVECs were scattered across the observable field, with very little visible development of luminal structures. It appeared that 200 µg/mL SeCS had the greatest effect on tube formation.

Moreover, the key parameters including node, junction and branch lengths were analyzed using ImageJ 1.48v software. The results are presented in Figure 2B–D. Statistically significant changes in nodes, junctions and branches were observed between the control and SeCS group (* *p* < 0.05). These results indicated that SeCS possessed anti-angiogenic activity. Moreover, the rate of lumen formation with regard to the number of branches (Figure 2D) of HUVECs with 200 µg/mL SeCS was 23.60 ± 9.94%, which was markedly lower than that for the CS group (93.57 ± 47.55%) and that of the SeNPs group (70.09 ± 23.07%). The results showed that the anti-angiogenic effect of 200 µg/mL SeCS was markedly greater than that of CS and SeNPs.

**Figure 3:**
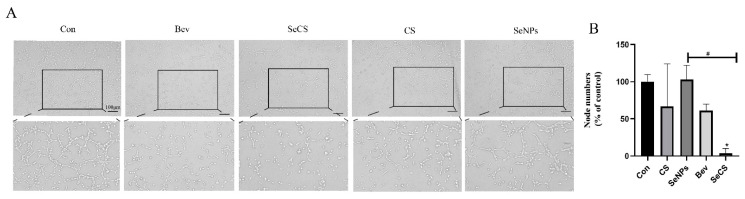
(**A**) HMEC-1 cells were subjected to tube formation assay with 200 μg/mL of CS, SeNPs, SeCS and Bev. (**B**) The generation rate for the number of primary nodes produced by different groups at a concentration of 200 µg/mL. * *p* < 0.05 compared to the control group, respectively. ^#^ *p* < 0.05 compared with each other of sample groups. Scale bars = 100 μm.

To verify the above results, we used HMEC-1 cells to carry out tube formation experiments, as detailed in Figure 3. The number of nodes of HMEC-1 cells with 200 µg/mL SeCS was 3.75 ± 6.33%, which was markedly lower than that for the SeNPs group (103.1 ± 18.98%), and the results were consistent with those of HUVECs (Figure 3B). Therefore, 200 µg/mL SeCS was used in the subsequent in vitro experiment.

### 2.2. Effect of SeCS on HUVECs Viability

**Figure 4:**
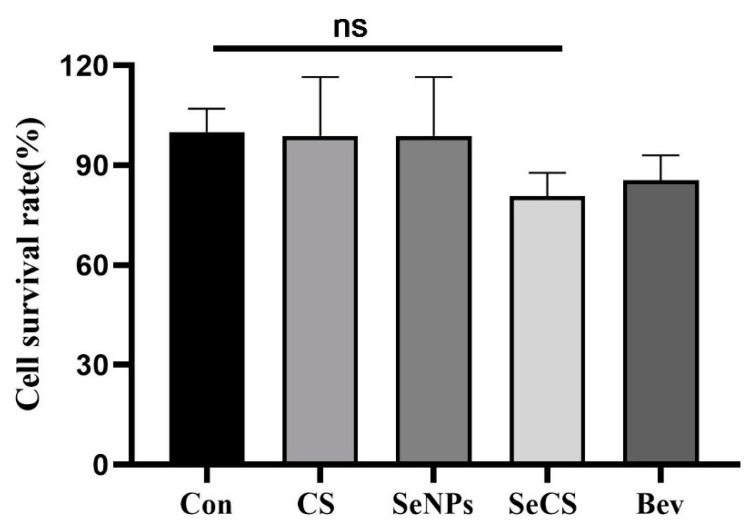
Effects of CS, SeNPs, SeCS and Bev at 200 µg/mL on the cell viability of HUVECs.

To detect whether the anti-angiogenesis of SeCS is associated with the inhibition of cell proliferation, the cell viability of HUVECs was measured using the CCK-8 assay [26]. HUVECs were treated with 200 µg/mL CS, SeNPs, SeCS and Bev for 24 h. The results are shown in Figure 4. Compared with the control group, the cell survival rate in the SeCS group decreased from 100 ± 7.07% to 80.67 ± 4.06%. Moreover, no significant difference in cell viability was observed among the different groups. The results indicated that the inhibitory effect of SeCS on HUVECs is negligible. Li et al. also showed that the effect of Au nanoclusters on angiogenesis was not achieved by inhibiting cell growth [27]. Hwang et al. showed that 1,8-Dihydroxy-3-methoxy-anthraquinone (DMA) was effective in inhibiting angiogenesis without any cytotoxicity when the concentration was as high as 20 μM. These data suggest that the anti-angiogenic ability of DMA is not due to its cytotoxic effects on cells. In contrast, DMA appears to single-handedly target the angiogenesis process while maintaining cell integrity [28]. Our results are similar to those in the literature mentioned above, but more experimental concentrations of SeCS are needed to confirm this result.

### 2.3. SeCS Inhibited HUVECs’ Migration

**Figure 5:**
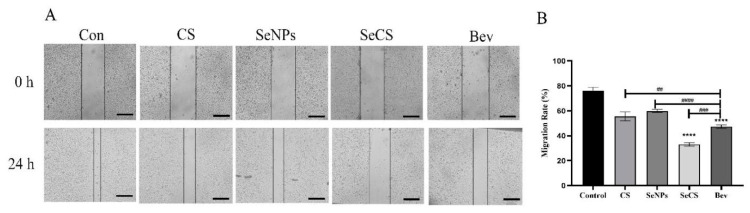
Effects of different treatments on wound healing ability of HUVECs. (**A**) Micrographs of scratched HUVECs treated with 200 μg/mL CS, SeNPs, SeCS and Bev for 0 and 24 h (200×). (**B**) Histogram of scratch healing rate of HUVECs. **** *p* < 0.0001 compared to the control group, respectively. ^##^ *p* < 0.01, ^###^ *p* < 0.001, and ^####^ *p* < 0.0001 compared with each of the other sample groups.

In order to investigate the effect of SeCS on the motility of HUVECs in vitro, we constructed a metastasis model in vitro. The wound healing test can evaluate the movement ability of cells in 2D space [12]. The results are shown in Figure 5. As Figure 5A shows, after cells were treated with CS, SeNPs, SeCS and Bev for 24 h, the gap left by scratches in the SeCS treatment group was larger than that in the CS and SeNPs treatment groups, indicating that the degree of wound healing of cells treated with SeCS was weaker than that of cells treated with CS and SeNPs. It is clear from Figure 5B that, after 24 h, the cell healing rate in the SeCS treatment group was 33.11 ± 0.81%. It was lower than that of the CS group (55.53 ± 2.02%) and SeNPs group (59.95 ± 0.72%), indicating that SeCS was stronger than CS and SeNPs in inhibiting cell wound healing. Wang et al. revealed that CS from Acipenser schrenckii had significant anti-proliferation and inhibition effects on scratch healing [29], which is consistent with the experimental results of this study. In conclusion, after the combination of CS and SeNPs to form SeCS, the ability to inhibit cell healing significantly increased.

### 2.4. SeCS Inhibited HUVECs Invasion

**Figure 6:**
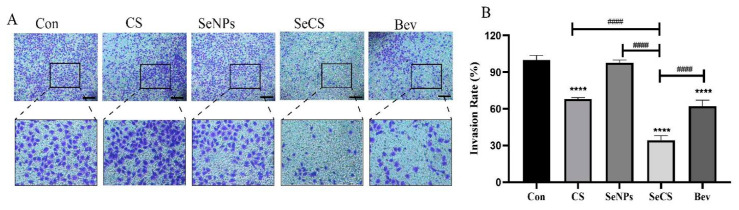
Effects of different treatments on invasion ability of HUVECs. (**A**) Invasion of HUVECs treated with 200 μg/mL CS, SeNPs, SeCS and Bev for 24 h (200×). (**B**) Histogram of HUVECs invasion rate. **** *p* < 0.0001 compared to the control group, respectively. ^####^ *p* < 0.0001 compared with each of the other sample groups.

The presence of matrix glue prevents the cells from migrating directly through the polyester carbonate membrane to the lower compartment, but only after the secretion of matrix metalloproteinase, which can degrade the matrix glue allowing it to be transferred through the polyester carbonate membrane to the lower compartment. Crystal violet can stain the cells migrating to the lower chamber blue–purple for observation and counting, and the number of cells migrating to the lower chamber may reflect the invasion ability of cells [30]. As Figure 6A shows, after CS and SeNPs treatment, more cells invaded than in the control group, but the effect is not obvious. And, after treating the cells with SeCS, the number of cells invading into the lower compartment was significantly reduced compared to CS and SeNPs, and the lower the number of cells invading the lower compartment suggests that SeCS diminished the ability of the cells to invade and penetrate the stromal gel. Moreover, the invasion rate of cells treated by SeCS was 34.42 ± 2.13%, which was significantly lower than that of 68.17 ± 0.60% in CS treatment group and the SeNPs treatment group attack rate of 97.74 ± 1.30% (Figure 6B). These results suggested that SeCS inhibited cellular invasion better than CS and SeNPs.

### 2.5. Effect of SeCS on CAM Neovascularization

**Figure 7:**
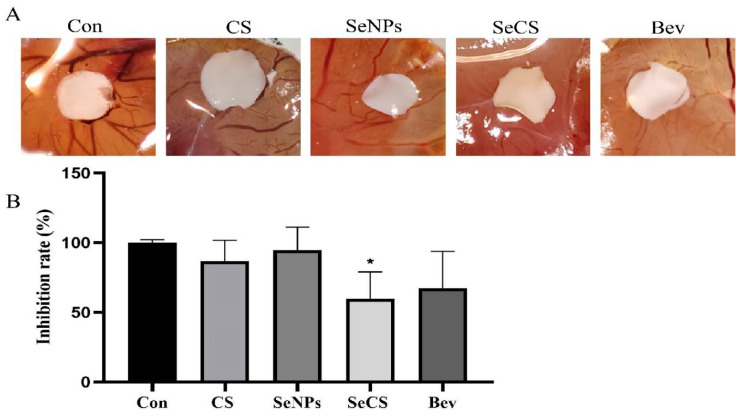
Effects of CS, SeNPs, SeCS and Bev at 200 µg/mL on the suppression of angiogenesis in a CAM model. (**A**) Images of CAMs treated with the same concentration for 48 h. (**B**) The rate of inhibition of chick embryo allantoic membrane angiogenesis (*n* = 50). * *p* < 0.05 compared to the control group, respectively.

In order to further explore the effect of SeCS on angiogenesis in vivo, we built a CAM angiogenesis model. During the eighth to tenth day of chick embryo development, a large number of blood vessels will be generated on the CAM on the inner surface of the eggshell membrane, and the immune system of the chick embryo will not yet be sound. This model can be used to study the influence of external substances on angiogenesis [31]. The results are shown in Figure 7A. In the control group, the CAM blood vessels displayed robust growth patterns, featuring leaf/vein-like shapes, radial distributions and well-defined structures with thick main vessels. Conversely, in the SeCS-treated group, the development of new capillaries was impeded, as indicated by a reduced vessel density, narrower diameter, disrupted structure and a lighter, blurry appearance. Additionally, there were fewer connected branches, and areas lacking vessels were noticeable. Notably, no “vascular convergence” or “vascular attraction” was observed [32]. The neovascularization rate in the SeCS group was 59.77 ± 9.66%, which was markedly lower than that in the control group (* *p* < 0.05) and lower than that in the CS group (86.82 ± 7.43%) and the SeNPs group (94.52 ± 8.31%) (Figure 7B). These results suggest a SeCS can significantly inhibit angiogenesis in the chick embryo allantoic membrane.

### 2.6. Effect of SeCS on Protein Expression in HUVECs

**Figure 8:**
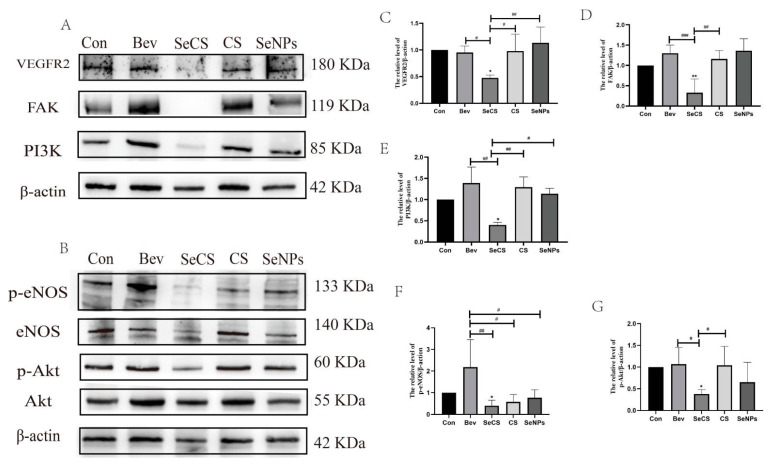
Expression levels of VEGFR2 and PI3K/Akt signaling pathway proteins by Western blot. (**A**) Band chart of VEGFR2, FAK and PI3K proteins expression. (**B**) Band chart of p-eNOS/eNOS and p-Akt/Akt proteins expression. (**C**–**G**) Bar charts of VEGFR2, FAK, PI3K, p-eNOS/eNOS and p-Akt/Akt protein expression (*n* = 3). * *p* < 0.05, ** *p* < 0.01 compared to the control group, respectively. ^#^ *p* < 0.05, ^##^ *p* < 0.01, ^###^ *p* < 0.001 compared with each of the other sample groups.

**Figure 9:**
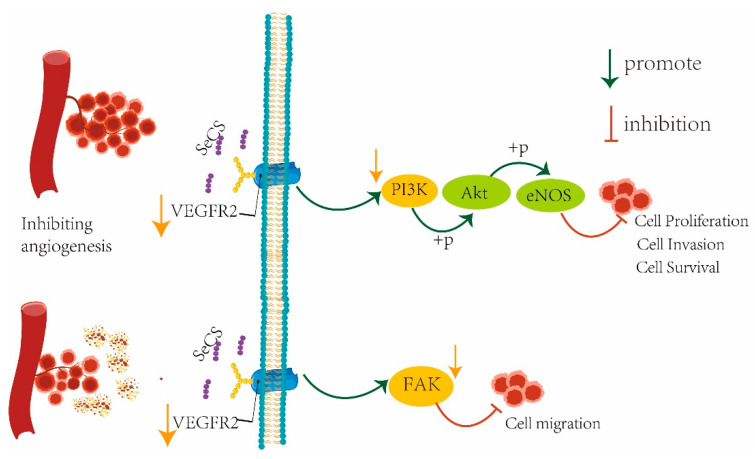
Signaling pathway diagram of SeCS inhibition of angiogenesis.

VEGFR2, a tyrosine protein kinase that acts as a cell surface receptor for VEGFA, VEGFC and VEGFD, plays an important role in the regulation of angiogenesis, vascular development, vascular permeability and embryonic hematopoiesis, and promotes endothelial cell proliferation, survival, migration and differentiation [33,34]. Tian et al. inhibited angiogenesis through the VEGF/VEGFR2 signaling pathway in their study, thereby inhibiting the growth and metastasis of breast cancer in in vivo and in vitro experiments [35]. The PI3K/Akt pathway is an intracellular signaling pathway that responds to extracellular signals to promote metabolism, proliferation, cell survival, growth and angiogenesis, a process that is mediated by the serine or threonine phosphorylation of a series of downstream substrates and involves key genes such as phosphatidylinositol 3-kinase (PI3K) and Akt protein kinase B [36]. The PI3K pathway is also known as the PI3K/Akt pathway, and PI3K is a key gene in the development of neovascularization and is regulated by lipid and protein phosphatases that control cell growth, proliferation and neovascularization [37]. FAK, one of the main target proteins downstream of VEGFR-2, is a tyrosine kinase that is usually involved in cell adhesion and motility. The inactivation of FAK is associated with the rapid dissolution of cell integrity in endothelial cells, the disintegration of cell adhesion molecules and the initiation of capillary formation [38]. Therefore, to analyze the anti-angiogenic mechanism, the role of SeCS in key protein expression in the VEGFR2-dependent and PI3K/Akt angiogenic pathway was analyzed through Western blotting. As Figure 8A,C,D,E show, compared with the CS- and SeNPs-treated groups, the protein bands of VEGFR2, FAK and PI3K became fainter after the cells were treated with SeCS, indicating that SeCS was able to significantly inhibit the down-regulation of the protein expression of VEGFR2, PI3K and FAK. Akt and eNOS are common carcinogenic proteins; phosphorylated Akt (p-Akt) and phosphorylated eNOS(p-eNOS) continue to participate in cell apoptosis and proliferation, and regulate a variety of biological processes, such as cell survival [39], and reducing their expression can effectively inhibit the formation of blood vessels [40]. As Figure 8B,F,G show, the Akt and eNOS protein levels of each target protein did not change significantly in normal cells, but the phosphorylation levels of Akt and eNOS proteins were significantly reduced after SeCS stimulation, which corresponds to the results in our study. The results indicate that SeCS downregulated the expression of VEGFR2, then further inhibited FAK and PI3K/Akt signaling pathway activation, which inhibited the migration, invasion and tube generate of HUVECs. On this basis, a signaling pathway diagram involving in the inhibitory effect of SeCS on angiogenesis was constructed, as shown in Figure 9.

## 3. Materials and Methods

### 3.1. Materials and Reagents

CS from shark cartilage (95% purity, Mw = 499.37, Catalog no. C107703) was obtained from Aladdin Reagent Co., Ltd. (Shanghai, China). CS consists of chondroitin 6-sulfate and chondroitin 4-sulfate, and the ratio of chondroitin 6-sulfate to chondroitin 4-sulfate measured by high performance liquid chromatography is equal to or greater than 0.33:1. Sodium selenite (Na_2_SeO_3_) with a purity ≥ 98% was obtained from Sigma Co., Ltd. (St. Louis, MO, USA). Ascorbic acid (VC) was sourced from Shanghai Yuanye Biotechnology Co., Ltd. (Shanghai, China). HUVECs and HMEC-1 cells were obtained from ATCC (Manassas, VA, USA) and Pricella Biotechnology Co., Ltd. (Wuhan, China), respectively. The matrix gel (Catalog no. 356234) was acquired from BD Bioscience (Pasadena, CA, USA). Dulbecco’s modified Eagle’s medium (DMEM) and trypsin-EDTA were obtained from Gibco Thermo Fisher Scientific Co., Ltd. (Shanghai, China). HMEC-1 cell-specific medium (Catalog no. CM-0576) was obtained from Pricella Biotechnology Co., Ltd. (Wuhan, China). Fetal bovine serum (FBS) was supplied by Procell Life Science & Technology Co., Ltd. (Wuhan, China). CCK8 was procured from Biosharp Biotechnology Co., Ltd. (Shanghai, China). The bevacizumab injection (Bev) was provided by Qilu Co., Ltd. (Jinan, China). Dimethyl sulfoxide (DMSO) was obtained from Eon Chemical Co., Ltd. (Shanghai, China). The bicinchoninic acid (BCA) kit and QuickBlock™ Blocking Buffer for Western blot were purchased from Beyotime Biotechnology Co., Ltd. (Shanghai, China). Primary antibodies targeting VEGFR2, PI3K, FAK, eNOS/p-eNOS, Akt/p-Akt and were purchased from Affinity Biosciences Co., Ltd. (Liyang, China). Chicken eggs were purchased from Xinxing Dahua Nong Poultry Egg Co., Ltd. (Yunfu, China). Other chemical reagents were of analytical grade.

### 3.2. SeCS Preparation

In accordance with the SeCS preparation method by Chen et al. [21], Na_2_SeO_3_ and VC with a molar mass ratio of 1:8 were dissolved in 30 mL CS at 0.1 mg/mL. The solution was stirred for 3 h at 25 °C, followed by 48 h dialysis with a membrane with an MW cutoff of 3500 against distilled water and freeze-drying to obtain SeCS. The color of the sample obtained was dark red. The particle size and selenium content of the SeCS were 131.3 ± 4.4 nm and 33.18%, respectively. After identification by FTIR, XRD, SEM and EDS, amorphous spherical nanoparticles with zero valence state SeCS were obtained. SeCS was mainly formed by physical adsorption between CS and SeNPs, and its surface elements were composed of 65.73% Se, 25.55% C and 7.44% O [20].

### 3.3. Cell Culture

HUVECs (fourth generation of primary cells) were cultured in DMEM supplemented with 10% bovine serum and 100 U/mL penicillin and 100 mg/mL streptomycin. HMEC-1 cells were cultured in HMEC-1 cell-specific medium. When the density of the cells reached 70–80%, the cells were passed down the generations and stored in an incubator (37 °C, 5% CO_2_), and the cells with good logarithmic growth were removed for subsequent detection.

### 3.4. Tube Formation Assay

We carried out a tube formation test to assess whether SeCS has a specific inhibitory impact on angiogenesis. Each well of the pre-chilled 96-well plates was coated with matrigel (50 µL) and then incubated for 30 min at 37 °C. Following the solidification of the matrigel, the HUVECs (fifth generation) were harvested from the T25 flasks, plated (2.0 × 10^5^ cells/well) in the gel with DMEM and allowed to grow for 6 h. Concurrently, the different concentrations of CS, SeNPs, SeCS and Bev (50, 100, 200, and 400 µg/mL) were added. The process of HMEC-1 cells (fourth generation) is the same as that of HUVECs, except that only 200 µg/mL of CS, SeNPs, SeCS, and Bev were added. After 6 h of groom, the generated vascular tubes were immobilized prior to imaging with an inverted light microscope. Imaging was performed using the Cytation 5 cell imaging multimode reader (Olympus SpinSR10 spinning disk confocal super-resolution microscope, Tokyo, Japan).

### 3.5. Cell Viability Assay

HUVECs (5000/well, fifth generation) were inoculated into 96-well plates. After 24 h, 200 µL of CS, SeNPs, SeCS and Bev were added to each well at specific doses. After 24 h of treatment, a CCK-8 assay was utilized to measure cell viability. Thereafter, CCK-8 reagent (10 µL) was added to each well for 3 h of incubation. Then, the absorbance was measured at 450 nm using a VarioskanFlash microplate reader (Thermo Fisher Scientific, Waltham, MA, USA).

### 3.6. Wound Healing Assay

We conducted a wound healing assay to analyze the proliferation capacity of SeCS [41]. The cells (8 × 10^4^/well, fifth generation) were inoculated into a 24-well plate and cultivated until approximately 80–90% confluence. A straight scratch was made using a 200 µL tip and photographed after 0 h. Then, the cells were incubated with 200 µg/mL CS, SeNPs, SeCS and Bev. The wounds were observed at 24 h and images were captured using a microscope. The migration rate was calculated as follows: migration rate (%) = (scratch distance at 0 h—scratch distance at 24 h)/scratch distance at 0 h × 100%.

### 3.7. Transwell Invasion Assay

Transwell invasion assays were conducted to assess the inhibitory effect of SeCS on HUVECs invasion. Specifically, a 24-well Transwell Boyden chamber equipped with inserts (6.5 mm in diameter) and a polycarbonate filter (8 µm in pore size) coated with matrigel matrix was utilized to analyze cell invasive ability. The cells (fifth generation) were subjected to 12 h of starvation prior to adjusting the cell density to 5.0 × 10^5^ cells/mL. Then, cells (200 µL) treated with CS, SeNPs, SeCS and Bev (200 µg/mL) were added to the upper chamber, whereas DMEM (750 µL) that contained 20% FBS was poured into the bottom chamber. Finally, the cells on the upper membrane were wiped with a cotton swab, and invading cells were fixed with methanol for crystal violet staining. An inverted microscope was used to obtain images. The invasion rate (%) was determined by the invading cell count of each group divided by the invading cell count of the control group × 100%.

### 3.8. Chicken Embryonic Chorioallantoic Membrane (CAM) Assay

A CAM assay was preformed to analyze the anti-angiogenic effect of SeCS. Chicken eggs were incubated for seven days at 37 °C and 50–65% relative humidity. Thereafter, a drill was utilized to make an artificial window (0.3–0.4 cm^2^) in the air sac to expose the chicken embryonic CAM. Thereafter, sterile qualitative filter paper disks (5 mm in diameter) that contained CS, SeNPs, SeCS and Bev (200 µg/mL) and PBS (20 µL per egg) were added to the CAM. Tape was used to cover the eggs for another 48 h incubation. In addition, the CAM was immersed in 4% paraformaldehyde, cut using a filter paper carrier with caution and utilized as the center for photography. ImageJ software was used to quantify new blood vessels on the CAM. The vascular area of each group was measured, and the percentage of vascular area relative to the total area of the CAM was calculated. The following formula was used: vascular neovascularization rate (%) = (area of vascular neovascularization at the eggshell opening site/area of the urothelium at the eggshell opening site)

### 3.9. Western Blot Assay

After HUVECs (fifth generation) were treated with CS, SeNPs, SeCS and Bev (200 µg/mL), total cellular proteins were extracted and separated through 10% sodium dodecyl sulfate-polyacrylamide gel electrophoresis (SDS-PAGE) prior to being transferred to 0.45 µm polyvinylidene fluoride (PVDF) membranes. The membranes were blocked with blocking buffer and then incubated overnight incubation using primary antibodies at a 1:500 dilution. The membranes were then washed with TBST, followed by further probing using an HRP-conjugated secondary antibody for a 2 h period. The protein bands were imaged with a Bio-Rad image analysis system (Bio-Rad Laboratories, Hercules, CA, USA). Moreover, ImageJ software was utilized to quantify the protein levels.

### 3.10. Statistical Analysis

Each experiment was performed at least three times. Quantitative data are presented as the means ± SD and were analyzed with GraphPad Prism 8.0. The means of two groups of data were compared using the unpaired, two-tailed Student’s *t*-test, whereas multiple groups were compared by one-way analysis of variance (ANOVA). *p* < 0.05 and *p* < 0.01 (or 0.001, 0.0001) indicate significance and extreme significance, respectively.

## 4. Conclusions

In conclusion, our study showed that SeCS was capable of suppressing angiogenesis created in both HUVECs and CAM. Further research revealed that SeCS inhibited the angiogenesis by reducing VEGFR2 expression and downstream PI3K/Akt signaling pathway. In summary, this study proved that SeCS possess the potential to be a promising candidate for anti-angiogenic drugs in the treatment of human cancers. However, the detailed molecular mechanisms and precise molecular targeting of the anti-angiogenic activity of SeCS require further experiments and its biological safety and efficacy need to be further validated if it is to be used as a safe anti-angiogenic drug.

## Data Availability

The data presented in this study are available on request from the corresponding author.
